# Supplementary material for: Machine learning photodynamics decode multiple singlet fission channels in pentacene crystal
Source: Nat Commun. 2025 Jan 30;16:1194. doi: 10.1038/s41467-025-56480-y (PMC11782655; doi:10.1038/s41467-025-56480-y)
Supplement: Supplementary file 1 — Supplementary Information [file 41467_2025_56480_MOESM1_ESM.pdf]

Machine learning photodynamics decode multiple singlet fission channels in pentacene crystal

Zhendong Li,[a†] Federico J. Hernández,[b†] Christian Salguero,[c] Steven A. Lopez,[c,*] Rachel Crespo-Otero,[b,*] and Jingbai Li[a,*]

[a] Hoffmann Institute of Advanced Materials, Shenzhen Polytechnic University, Shenzhen, 518055, People's Republic of China
[b] Department of Chemistry, University of College London, London, WC1H0AJ, U.K.
[c] Department of Chemistry and Chemical Biology, Northeastern University, Boston, 02115, U.S.A.
†These authors contributed to this work equally

* Correspondence authors and emails:
Steven A. Lopez: s.lopez@northeastern.edu
Rachel Crespo-Otero: r.crespo-otero@ucl.ac.uk
Jingbai Li: lijingbai@szpu.edu.cn

Table of Content

Supplementary Note 1. Dimer models 2
Supplementary Note 2. Quantum mechanical calculations 2
Supplementary Note 3. Neural network training 7
Supplementary Note 4. Simulated absorption spectra 9
Supplementary Note 5. ML photodynamics simulations 11
Supplementary Note 6. Intermolecular vibrations 13
Supplementary Note 7. Minimum energy conical intersections 14
Supplementary Note 8. Rigid potential energy scans 15
Supplementary Note 9. Notes on the crystal environment 19
Supplementary Note 10. Data and code availability 20
Supplementary reference 20

Supplementary Note 1. Dimer models

Here, we briefly discuss the validity of the dimer models for studying the excitons in the pentacene crystal. Sharifzadeh and co-workers reported an average electron-hole distance for the singlet excited states of 6–8Å.[1] Supplementary Figure 1 shows the intermolecular distances between the monomers.

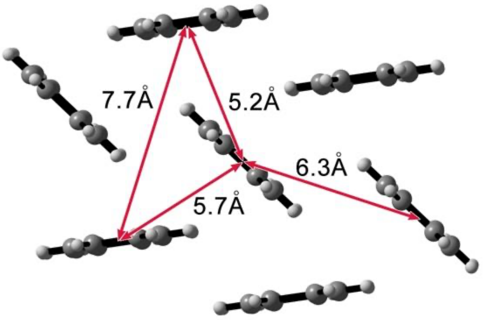

Supplementary Figure 1. Side view of the pentacene crystal. The intermolecular distances are highlighted with red arrows.

The dimer model can describe the charge transfer (CT) reported at 6 Å, while computing the CT at 8 Å requires a tetramer model. They also reported the average electron-hole distances of 2 Å in the triplet state.[1] It suggests the SF will rapidly collapse the delocalized singlet exciton to a localized triplet pair state, as reported by Miller and co-workers.[2] Neaton and co-workers show clear singlet exciton wavefunctions, where the electrons mainly delocalized over the monomers neighboring to the hole on the central pentacene.[3] Thus, the dimer model could capture most CT characters of the singlet exciton. Given the excellent agreement in the SF time constants between the reference (30-70 fs) and our works (33-61 fs), we think the dimer model reasonably approximates the SF processes of pentacene crystal.

Supplementary Note 2. Quantum mechanical calculations

We performed quantum mechanical (QM) calculations to compute the geometries and excitation energies of the pentacene dimers. We optimized the pentacene dimers in the crystal models to the ground state minimum with the two-layer electrostatic embedding ONIOM calculations implemented in fromage.[4] The QM part calculations and the RESP change calculations employ the ωB97XD/def2-TZVP methods implemented in Gaussian 16.[5] The energies of the entire crystal structures are computed by the GFN2-xTB method in xTB 6.5.1.[6] The ground-state vibrational modes and frequencies are computed analytically using the same method which shows all positive frequencies. To optimize the excited-state minima and minimum energy conical intersections (MECIs), we switch to the SA6-CASSCF(4,4)/ANO-S-VDZP calculations available in OpenMolcas 19.11.[7] The NN training data are computed with the BAGEL program,[8] which achieves better parallel efficiency than OpenMolcas in our tests (Supplementary Table 1). Since the ANO-S-VDZP basis sets are unavailable in BAGEL, we choose the comparable cc-pVDZ basis set that produces consistent results in the SA6-CASSCF(4,4) calculations for the pentacene dimers. The training data calculations embedded the constant background charges as we used in the two-layer ee-ONIOM(SA6-CASSCF(4,4)/cc-pVDZ:GFN2-xTB) calculation, where the QM part was used to train NN. The excited-state vibrational modes and frequencies are computed with the SA6-CASSCF(4,4)/cc-pVDZ level using the numerical differentiation approach. Supplementary Figure 2 collects the active orbitals in the (4,4) space of the herringbone and parallel dimers. In the machine learning (ML) photodynamics simulations, the QM calculations in the two-layer electrostatic

embedding ONIOM approach are replaced with the NN predictions, which are implemented in PyRAI^{2}MD.[9]

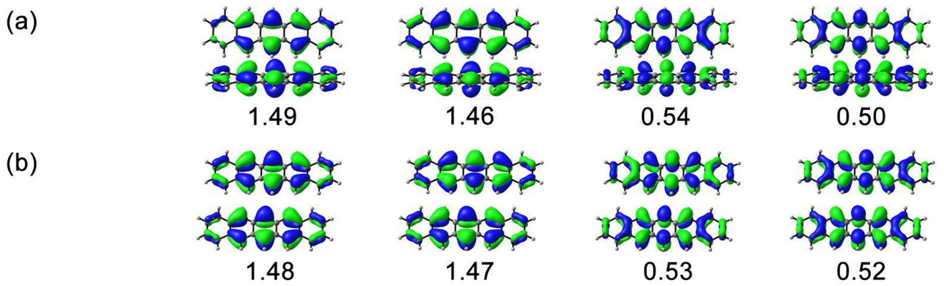

Supplementary Figure 2. Active orbitals for the pentancene dimers. (a) herringbone and (b) parallel dimer with the occupation numbers computed at the SA6-CASSCF(4,4)/cc-pVDZ level.

The CASSCF calculations often overestimate the excitation energies due to the lack of electron dynamical correlations. A previous study by Zimmerman and co-workers showed the CASSCF method can properly describe the doubly-excited states and multi-exciton states.[10] However, we found no direct benchmarks of the SA6-CASSCF(4,4)/cc-pVDZ calculations against the multireference methods, such as XMS-CASPT2. As it is essential to learn the dynamical correlation in the excited-state potential energy surface, we computed the XMS6-CASPT2(4,4)/cc-pVDZ energies to quantify the role of dynamical correlation compared to the CASSCF potential energy curves. To reduce the limitation of the selected active space, we computed the potential energies using the mixed-reference spin-flip time-dependent density functional theory (MRSF-TDDFT),[11] which can generate spin-adapted multireference excited-state wavefunction including dynamical correlations. The MRSF-TDDFT calculations used PBE0 functional, given its good accuracy in recent benchmarks.[12] Moreover, we computed the CASSCF energies using the (8,8) active spaces reported by Zimmerman and co-workers[13] to determine the influence of the active space on the potential energy curves. Supplementary Figure 3 illustrates the potential energy curves of the pentacene dimers computed with the XMS6-CASPT2(4,4)/cc-pVDZ, MRSF-TDDFT(PBE0)/cc-pVDZ, and SA6-CASSCF(8,8)/cc-pVDZ methods.

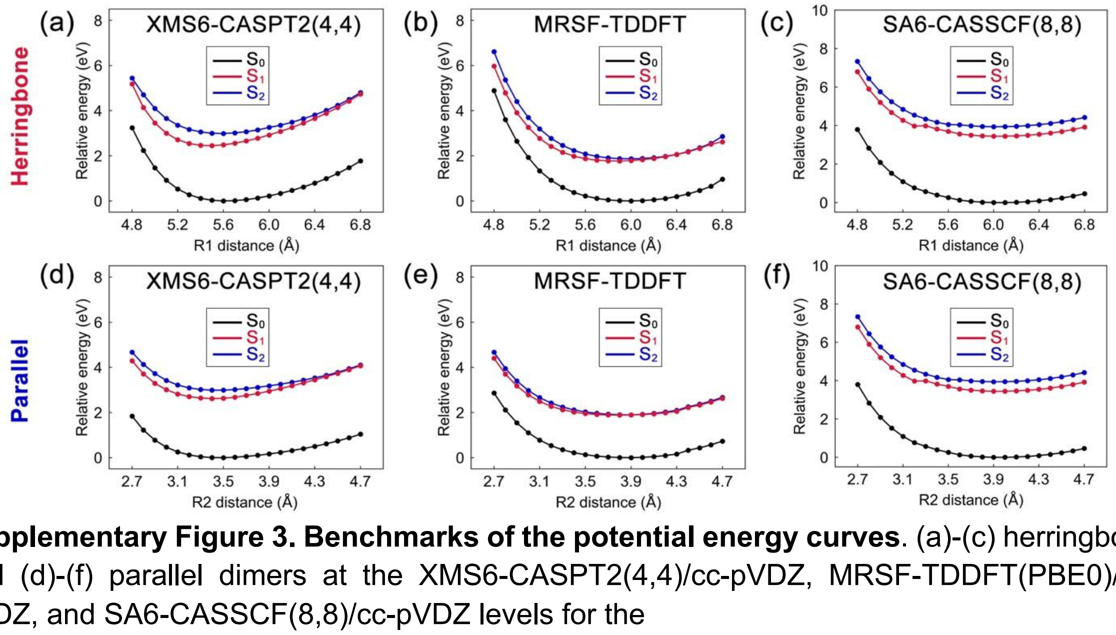

Supplementary Figure 3. Benchmarks of the potential energy curves. (a)-(c) herringbone and (d)-(f) parallel dimers at the XMS6-CASPT2(4,4)/cc-pVDZ, MRSF-TDDFT(PBE0)/cc-pVDZ, and SA6-CASSCF(8,8)/cc-pVDZ levels for the

The ωB97XD/def2-TZVP calculations optimized the S_0 minima of herringbone and parallel dimers at R1 = 5.84 Å and R2 = 3.70 Å. The XMS6-CASPT2(4,4)/cc-pVDZ calculations show underestimated distances at R1 = 5.6 Å and R2 = 3.5 Å. Although the dynamical correlation lowers the excited-state energies, the values (herringbone: 2.48 eV; parallel: 2.63 eV) are still higher than the experimental values of the pentacene crystal (1.83 eV).[13] These inconsistent results suggest that the XMS6-CASPT2(4,4)/cc-pVDZ method cannot capture all essential dynamical correlations, possibly due to the limited active space. The MRSF-TDDFT(PBE0)/cc-pVDZ calculations show the S_0 minima at R1 = 6.1 Å and R2 = 3.9 Å. The resulting vertical excitations are 1.79 eV and 1.89 eV for the herringbone and parallel dimers, close to the experimental value. Therefore, the MRSF-TDDFT(PBE0)/cc-pVDZ results seem a good reference for excitation energies. The SA6-CASSCF(8,8)/cc-pVDZ calculations show notably larger S_2-S_1 gaps than that with the (4,4) space, which overestimates the S_2-S_1 gaps compared to the MRSF-TDDFT(PBE0)/cc-pVDZ reference. Thus, the SA6-CASSCF(8,8)/cc-pVDZ results must be corrected by including the dynamical electron corrections.

The SA6-CASSCF(4,4)/cc-pVDZ calculations show S_0 minima at 6.1 Å and 4.0 Å for the herringbone and parallel dimers, which are in line with the MRSF-TDDFT(PBE0)/cc-pVDZ results but 0.5 Å longer than the XMS6-CASPT2(4,4)/ANO-S-VDZP results. These differences do not significantly affect the predicted SF time constants because the molecular vibrations (e.g., Wigner sampling) lead to more than 1.0 Å variations in the intermolecular distances according to the trajectories in Figure 3e, 3i, 3g, and 3k. The S_2/S_1 gap is essential to the SF time constants. The SA6-CASSCF(4,4)/cc-pVDZ results showed small S_2/S_1 gaps. The XMS6-CASPT2(4,4)/ANO-S-VDZP results showed a slightly larger S_2/S_1 gap (0.65 eV) in the herringbone dimer with R1 = 5.1 Å than the SA6-CASSCF(4,4)/cc-pVDZ results (0.34 eV). We do not expect this deviation to largely affect the SF time constants because the rest of the potential energy surface covered by the Wigner sampling (5.3–6.3Å) shows sufficiently small S_2/S_1 gaps (e.g., < 0.6 eV) for SF.

We computed the S_0-S_1 and S_0-S_2 electron density differences in the herringbone dimers to further compared SA6-CASSCF(4,4)/cc-pVDZ, XMS6-CASPT2(4,4)/ANO-S-VDZP and MRSF-TDDFT(PBE0)/cc-pVDZ calculations. The density difference $\Delta\rho$ is approximated by the sum of the squared norm of the wavefunction weighted by the overall changes of the orbital populations, derived from the electron density difference approach:[14]

$$\Delta\rho_i = \sum_j w_{i,j} \sum_k (N_{i,j,k} - N_{0,j,k})|\phi_k|^2$$

The subscripts *i*, *j*, *and k* denote the index of excited states, electronic configurations, and molecular orbitals. *w* is the weight of the configuration, *N* is the orbital occupation number, and ϕ is the molecular orbital.

The SA6-CASSCF(4,4)/cc-pVDZ results (Supplementary Figure 4a) show CT characters in S_1 and DE characters in S_2 at R1 = 4.8 Å. When R1 elongates, S_1 shows mixed CT and DE characters. We note that S_2 becomes local excitation (LE) at R1 > 6.1 Å. Supplementary Figure 4b shows the same S_1 nature at the XMS6-CASPT2(4,4)/ANO-S-VDZP level as that at the SA6-CASSCF(4,4)/cc-pVDZ level. The S_2 maintained the DE characters when R1 > 6.1Å at the XMS6-CASPT2(4,4)/ANO-S-VDZP level, suggesting underestimated DE characters at the SA6-CASSCF(4,4)/cc-pVDZ due to the lack of dynamical electron correlation. Nevertheless,

the agreement in the S_1 nature ensures the correct electronic configuration analysis, as shown in Figure 5. The MRSF-TDDFT(PBE0)/cc-pVDZ results in Supplementary Figure 4c are comparable to the XMS6-CASPT2(4,4)/ANO-S-VDZP results when R1 <6.1Å. It overestimated the CT characters in S_1 and underestimated the DE characters in S_2 when R1 > 6.1Å. This problem is caused by the incomplete double configurations generated by flipping a spin of one electron in a triplet reference. Therefore, we only considered the MRSF-TDDFT(PBE0)/cc-pVDZ results as a supplementary reference of the PES and S_2/S_1 gaps, but not for electronic structure analysis.

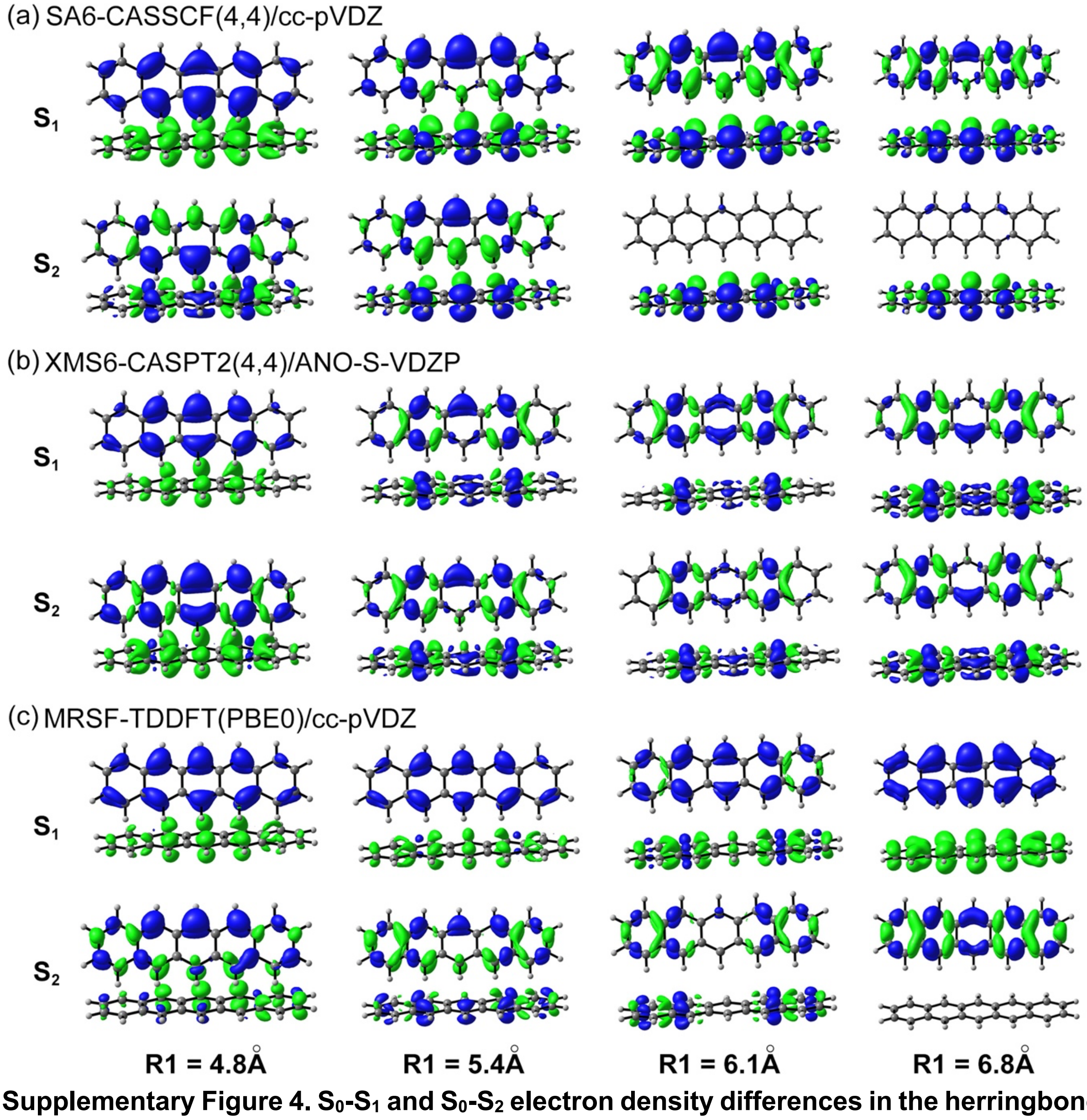

Supplementary Figure 4. S_0-S_1 and S_0-S_2 electron density differences in the herringbone dimers. (a) SA6-CASSCF(4,4)/cc-pVDZ, (b) XMS6-CASPT2(4,4)/ANO-S-VDZP, and (c) MRSF-TDDFT(PBE0)/cc-pVDZ. Blue and green refer to the depletion and accumulation of the electrons.

Supplementary Figure 5 illustrates the S_0-S_1 and S_0-S_2 electron density differences in the parallel dimers computed with the SA6-CASSCF(4,4)/cc-pVDZ, XMS6-CASPT2(4,4)/ANO-S-VDZP, and MRSF-TDDFT(PBE0)/cc-pVDZ methods. The SA6-CASSCF(4,4)/cc-pVDZ and XMS6-CASPT2(4,4)/ANO-S-VDZP results in Supplementary Figure 5a and S5b show good

agreement in the mixed CT and DE characters in the S_0-S_1 and S_0-S_2 electron density differences. The MRSF-TDDFT(PBE0)/cc-pVDZ calculations show consistent results when R2 < 4.7Å in Supplementary Figure 5c. As the distance increases, the missing double configurations underestimated the DE characters in S_1 and overestimated CT characters in S_2.

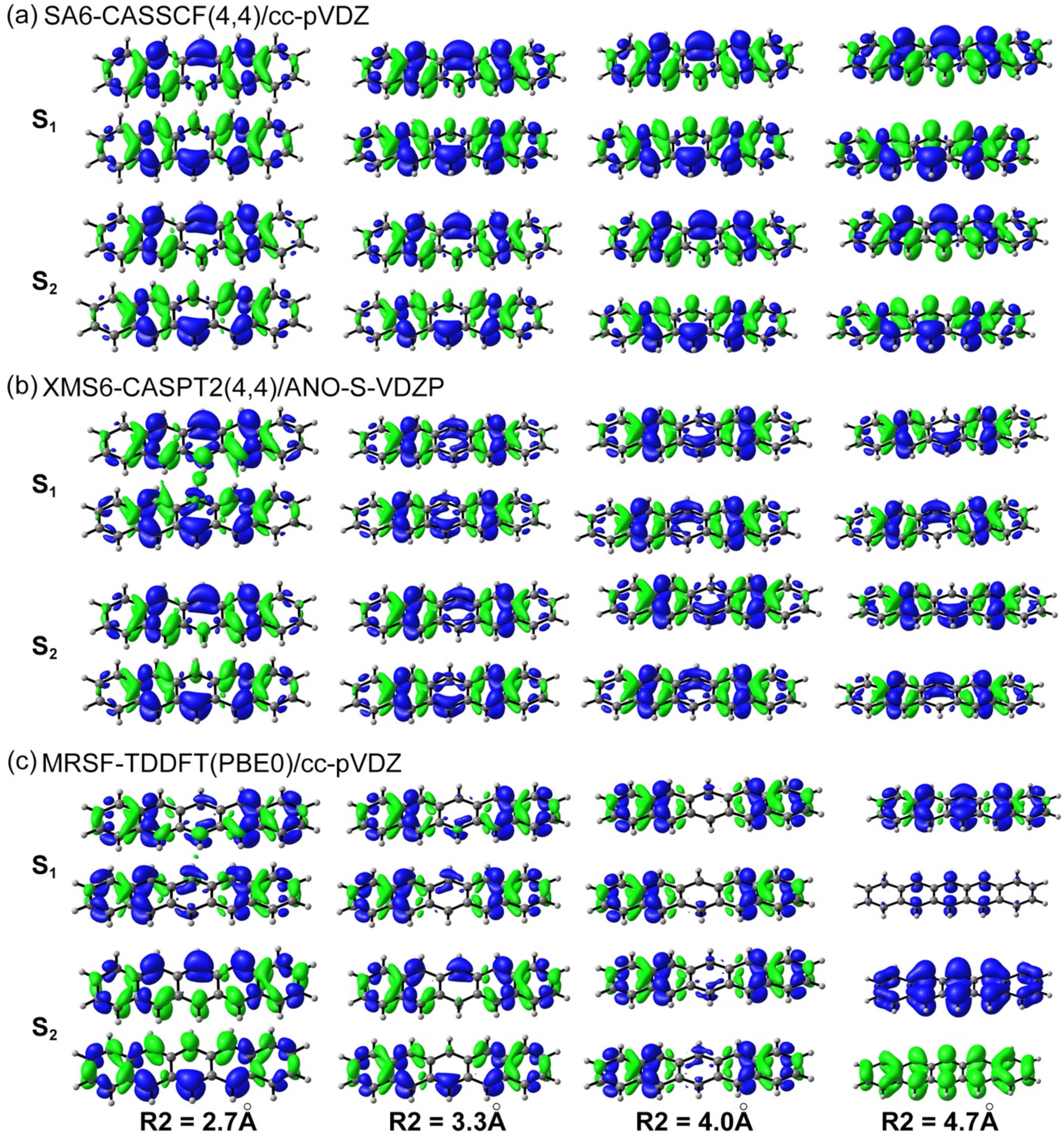

Supplementary Figure 5. S_0-S_1 and S_0-S_2 electron density differences in the parallel dimers. (a) SA6-CASSCF(4,4)/cc-pVDZ, (b) XMS6-CASPT2(4,4)/ANO-S-VDZP, and (c) MRSF-TDDFT(PBE0)/cc-pVDZ calculations. Blue and green refer to the depletion and accumulation of the electrons.

Collectively, our benchmarks show that SA6-CASSCF(4,4)/cc-pVDZ calculations produced consistent S_1 nature with the XMS6-CASPT2(4,4)/ANO-S-VDZP results and consistent PES and S_2/S_1 with the MRSF-TDDFT(PBE0)/cc-pVDZ results. Given the excellent agreement

between the predicted and experimental SF time constants, the SA6-CASSCF(4,4)/cc-pVDZ is an appropriate method for studying the SF mechanism of pentacene dimers in crystals.

Supplementary Table 1 lists the averaged computational costs for the single-point calculations using the above methods. We performed the SA6-CASSCF(4,4)/cc-pVDZ calculations using BAGEL, which were efficiently parallelized with 4 CPUs. The XMS6-CASPT2(4,4)/cc-pVDZ calculations with BAGEL failed because they require more than 500 GB RAM for a single calculation. Thus, we switched to OpenMolcas for the XMS6-CASPT2(4,4)/cc-pVDZ calculations, accelerated with 20 CPUs. The MRSF-TDDFT(PBE0)/cc-pVDZ calculations used the underdeveloped code, OpenQP,[12] paralleled with 4 CPUs.

Supplementary Table 1. Computational costs for single-point calculations of pentacene dimers.

Method	Time (s)	with gradient	CPUs
SA6-CASSCF(4,4)/cc-pVDZ	5205	no	4
SA6-CASSCF(4,4)/cc-pVDZ	9032	yes	4
XMS6-CASPT2(4,4)/cc-pVDZ	92032	no	20
MRSF-TDDFT(PBE0)/cc-pVDZ	2295	no	4
MRSF-TDDFT(PBE0)/cc-pVDZ	5090	yes	4

Computing the energy and gradient of one state at the SA6-CASPT2(4,4)/cc-pVDZ level costs 1.7 times higher than the energy-only calculation. The XMS6-CASPT2(4,4)/cc-pVDZ energy calculation requires more than 1 day, which is about 18 times longer than the SA6-CASSCF(4,4)/cc-pVDZ calculation. The gradient calculations at the XMS6-CASPT2(4,4)/cc-pVDZ exceed our affordable computational resources. The MRSF-TDDFT(PBE0)/cc-pVDZ calculations show promising efficiency, where the energy calculation without and with gradients only took 44% and 56% of the time spent by the SA6-CASSCF(4,4)/cc-pVDZ calculations. The efficiency of the MRSF-TDDFT(PBE0)/cc-pVDZ calculations is realized by the newly designed quantum chemistry code, OpenQP,[12] which was not released but only available for benchmark purposes while we wrote the manuscript. The OpenQP has now been formally published after the revision of our manuscript. The electrostatic embedding version of MRSF-TDDFT is still under development. Overall, the SA6-CASSCF(4,4)/cc-pVDZ shows a good balance between accuracy and efficiency.

Supplementary Note 3. Neural network training

We implemented the feed-forward neural network (NN) with multiple perceptron layers based on TensorFlow/Keras API for Python.[15] The NN computes the inverse distance matrix of the input molecule to predict the energies and gradients. The predicted energy gap between two electronic states is used to evaluate the curvature-approximated time derivative coupling (*k*TDC),[16-17] derived from the Baeck-An approximation.[18]

The NN employs a leaky softplus activation function. The energies and forces are trained with a combined loss function to ensure their physical relationship. The loss function is defined as

Loss = $w_E \cdot \sum(E_{pred} - E_0)^2/N + w_F \cdot \sum(F_{pred} - F_0)^2/(3N)$, where N is the number of atoms. The NN-predicted gradients are obtained from the analytical gradients of the NN. We use a learning rate scheduler that reduces the learning rate from 10^{-3} to 10^{-4} and 10^{-5} when the validation loss reaches a plateau. The training data was split into training and validation sets in a 9:1 ratio.

The hyperparameters of the NN potential are optimized with a grid search over the number of hidden layers (3, 4, 5, 6, 7, 8, 9, 10), nodes per hidden layer (200, 300, 400, 500, 600, 700), batch sizes (64, 128) and L2 regularization factors (10^{-7}, 10^{-8}, 10^{-9} and 10^{-10}). Supplementary Table 2 summarizes the final data size, training settings, and optimized hyperparameters. Supplementary Table 3 shows the NN prediction accuracy of energies and forces after the adaptive sampling.

Supplementary Table 2. Training setup and selected hyperparameters of the NN potentials.

System	Herringbone		Parallel	
Data size	4421		3455	
Activation function	Leaky softplus			
Train: Validation	9:1			
w_E: w_F	1:1			
Model	NN1	NN2	NN2	NN3
Layers	3	6	4	6
Nodes	700	500	700	600
Batch size	64	64	64	64
L2	10^{-10}	10^{-8}	10^{-9}	10^{-8}
Steps for $lr = 10^{-3}$	200			
Steps for $lr = 10^{-4}$	150			
Steps for $lr = 10^{-5}$	50			

The training data generation is mainly based on the Wigner sampling because the singlet fission (SF) of pentacene dimers occurs near the S_2 and S_1 Franck-Condon (FC) regions. We first generate 1000 non-equilibrium geometries for each dimer according to the vibrational frequencies and modes at the zero-point level. To enhance the learnability of the data, we added another 2000 structures by rescaling the atomic displacements in all vibrational modes to 90% and 80% in the Wigner sampling. The training data contain the energies and gradients of the lowest 6 singlet states. We use adaptive sampling to expand the initial training sets. We trained two sets of NNs as a committee model to propagate 100 trajectories from the S_2-FC points for 400 fs with a step size of 0.5 fs. The standard deviation (STD) in the predicted energy and gradients of the NN committee is used to measure the uncertainty of the current prediction. The trajectories are stopped when the STD exceeds the empirical thresholds for energy (0.03 Hartree) or gradients (0.12 Hartree·Bohr^{-1}), respectively. The last geometries of the stopped trajectories are collected and recomputed with the SA6-CASSCF(4,4)/cc-pVDZ calculations,

including the charges of the crystal shell. The recomputed data are added to the initial training set to retrain the committee model. After that, the adaptive sampling restarts the trajectories for the next iteration. To speed up the adaptive sampling, the trajectories were propagated in the gas phase with only the charges of the crystal shell. Supplementary Figure 6 plots the average simulation times and the number of collected structures as a function of iterations in the adaptive sampling for the pentacene dimers.

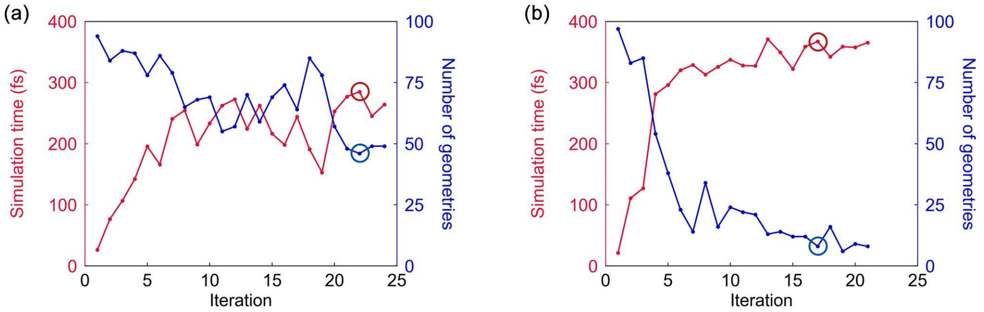

Supplementary Figure 6. Summary of adaptive sampling. Plots of the number collected geometries and average simulation time as a function of adaptive sampling iterations for (a) the herringbone and (b) parallel dimers, respectively. The red and blue circles mark the simulation time and number of sampled geometries at the 22 and 17 iterations for herringbone and parallel dimers, respectively.

We choose the NNs after 22 and 17 steps for the herringbone and the parallel dimer as their average simulation time achieves the maxima, and the number of uncertain structures reaches the lowest value at the same time. The final training sets increased to 4211 and 3455 data points for the herringbone and parallel dimers, respectively. Supplementary Table 3 list the final mean-absolute-error (MAE) and coefficient of determination (R^2) of the NNs.

Supplementary Table 3. The validation MAE and R^2 in the NN-predicted energies and gradients.

System	Herringbone		Parallel	
Model	NN1	NN2	NN1	NN2
MAE_{Energy}	0.0336	0.0363	0.0351	0.0421
R^2_{Energy}	0.9990	0.9991	0.9990	0.9987
MAE_{Grad}	0.1761	0.1974	0.1727	0.1825
R^2_{Grad}	0.9865	0.9842	0.9862	0.9854

Supplementary Note 4. Simulated absorption spectra

We simulated the absorption spectra of the pentacene dimers based on 1000 non-equilibrium geometries generated by Wigner sampling at the zero-point energy level. The wavelengths and intensities are obtained from the energies and oscillator strengths of the adiabatic electronic states, S_1-S_5.

The lowest optical bright absorption band (i.e., intensity > 0) is S_2 in both herringbone and parallel dimers. The counter-intuitive higher S_1 adsorption bands than S_2 results from the large

optical dark regions (i.e., intensity = 0) of the S_1 band. Supplementary Figure 5 plots the S_1 and S_2 wavelength distributions with absorption bands.

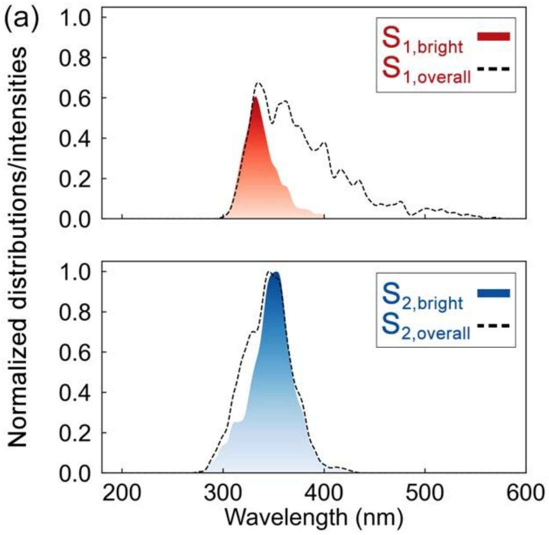

Supplementary Figure 7. **Plots of the S_1 and S_2 wavelength distributions with the adsorption bands, computed at the SA6-CASSCF(4,4)/cc-pVDZ level.** The overall distributions (dashed lines) correspond to the absorption of S_1 and S_2 with equal oscillator strengths, which are normalized to the maximum value of the S_2 curves. The bright part distributions (colored regions) are the normal absorption of S_1 and S_2 normalized to the maximum intensities of the S_2 band.

In both dimers, the wavelengths of S_1 are longer than S_2 in the same geometry. Most low-lying S_1 wavelengths have almost zero intensities, while most S_2 wavelengths show significantly higher intensities. As such, the absorption spectra show the S_1 adsorption band is higher than S_2, making S_2 the lowest accessible state by photoexcitation.

To understand the small transition-allowed region in the S_1 absorption band, we plot the oscillator strengths against the CSF weights in Supplementary Figure 8.

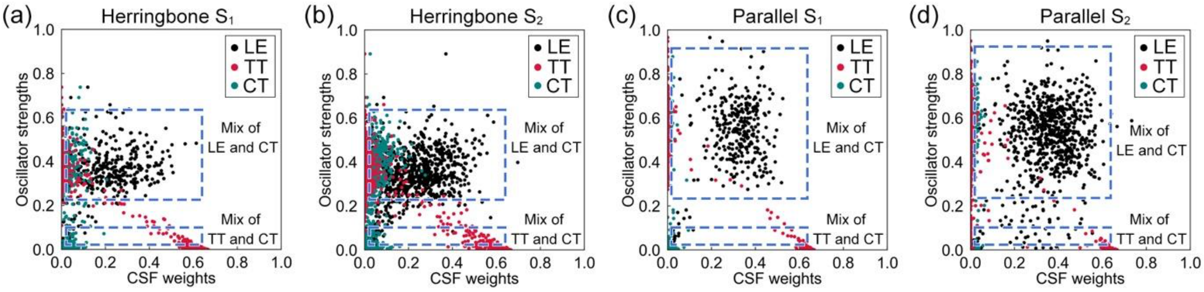

Supplementary Figure 8. Plots of oscillator strengths as functions of the CSF weights computed with the SA6-CASCI(4,4)/cc-pVDZ calculations. (a) S_1 and (b) S_2 states of the herringbone dimers and the (c) S_1 and (d) S_2 of the parallel dimer.

Supplementary Figure 8 shows negative correlations between the TT characters and the oscillator strengths in both dimers. It confirms that the adiabatic excited state becomes “dark” with a large TT character. The local excitation (LE) characters of each monomer display strong oscillator strengths in both S_1 and S_2, which are responsible for the absorption band observed in the simulated spectra. The CT characters show no correlation to the oscillator strengths, but these configurations mix with the TT and LE. Both herringbone and parallel dimers show

stronger oscillator strengths in S_2 because of their greater LE weights than in S_1. Therefore, the S_2 absorption bands show larger intensities than S_1 in the simulated spectra. The subsequent $S_2 \rightarrow S_1$ decay converts the LE characters to TT characters via mixing with the CT configurations. Therefore, our ML-photodynamics simulations from the S_2-FC region inform the CT-mediated SF pathways.

On the other hand, the mixing of LE and TT mediated with CT already brings small transition-allowed regions in S_1. These results agree with the proposed coherent SF pathway via direct excitation to the dark TT state,[19] which involved a charge resonance (CR) state (i.e., a symmetric superposition of the CT states) carrying finite transition probability borrowed from the main excitonic excitations. Note that the main excitonic excitation defined in this reference is equivalent to LE in our CSF representations. As such, our ML-photodynamics simulations from the transition-allowed S_1-FC region elucidate the coherent SF pathways. Collectively, our results suggest the coexistence of CT-mediated and coherent SF pathways.

In addition, Supplementary Figures8a and S8b show a larger mixing of CT and LE in the herringbone dimer than in the parallel dimer, as shown in Supplementary Figures8c and 8d. It suggests the direct excitation of the TT state could also be anisotropic according to the direction of the pentacene dimers.

Supplementary Note 5. ML photodynamics simulations

In the ML photodynamics simulations, we first propagated 1000 trajectories in the microcanonical ensemble (NVE) for the pentacene dimers from the S_2-FC points (termed S_2 trajectories). We considered a transition-allowed S_1-FC region with $\lambda_{S1} < 350$ nm according to Figure 2, where we propagated 330 and 339 trajectories for the herringbone and parallel dimers, respectively (termed S_1 trajectories). The surface hopping probability is computed using Tully's fewest switches surface hopping (FSSH) algorithm.[20-21] The non-adiabatic couplings are evaluated using the curvature-approximated time-derivative coupling (*k*TDC)[16-17] based on the NN-predicted energies. To determine the energy gap for *k*TDC calculation, we investigated the distributions of the S_2/S_1 surface hopping energy gaps with 100 trajectories of the photodynamics simulations with explicitly computed energies, forces, and NACs at the ee-ONIOM(SA6-CASSCF(4,4)/cc-pVDZ:GFN2-xTB) level. Supplementary Figures9a and S9b show the majority of the S_2/S_1 surface hops occurred with an energy gap < 0.1 eV. The average hopping energy gaps are 0.13 and 0.09 eV for the herringbone and parallel dimers, respectively. Supplementary Figure 9c plots the final S_2 population in the 50 fs ML-photodynamic simulations with the energy gap threshold from 0.5 to 0.05 eV. We find the best agreement with the experimental values when the energy gap is 0.1 eV. Thus, we chose a threshold of 0.1 eV for computing the *k*TDC in our 200 fs ML-photodynamics simulations.

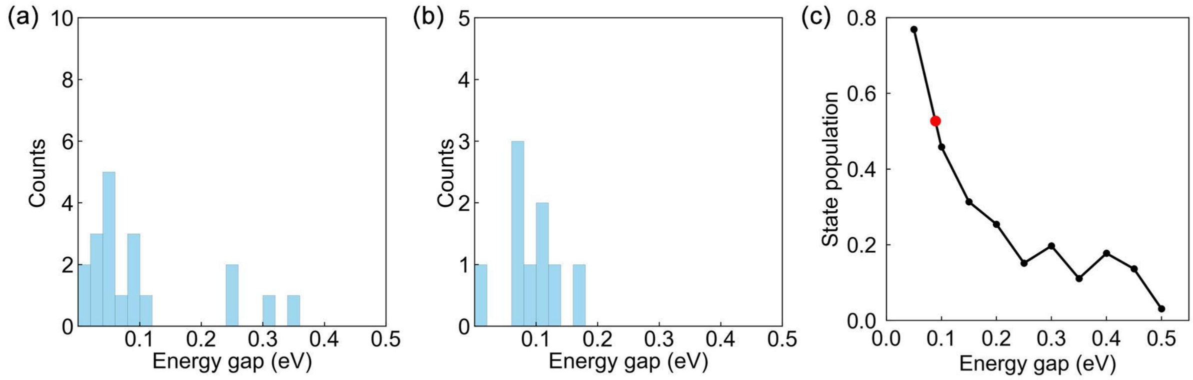

Supplementary Figure 9. **Distributions of the S_2/S_1 surface hopping energy gaps.** (a) the herringbone and (b) parallel trajectories at the ee-ONIOM(SA6-CASSCF(4,4)/cc-pVDZ:GFN2-xTB) level. The final S_2 population of the herringbone dimers in the 50 fs ML photodynamics simulations as a function of the energy gap threshold. The red dot marks the final S_2 population according to the experimental time constant of 78 fs.

We recomputed the electronic structures of the final snapshots with ee-ONIOM(SA6-CASCI/cc-pVDZ:GFN2-xTB) for all trajectories. Supplementary Table 4 lists the average and standard deviation of the weights for the TT states in the final snapshots.

Supplementary Table 4. Average weights of the TT characters in the S_1 state in the final snapshot of the ML-photodynamics simulations for herringbone and parallel dimers.

Dimer	Initial state	average TT weights	standard deviation
Herringbone	S_2	0.67	0.0028
	S_1	0.67	0.0009
Parallel	S_2	0.67	0.0010
	S_1	0.67	0.0010

Our SA6-CASCI(4,4)/cc-pVDZ calculations show that the TT state dominated the S_1 state at the end of the simulations from the S_2-FC points and the transition-allowed S_1-FC points. The average weight of 0.67 reached the maximum value we observed in the intermolecular distance scan (Figures 4e and 4f). The minor standard deviations imply all trajectories arrived at the TT states. Therefore, the $S_2 \rightarrow S_1$ decay time constants reflect the CT-mediated singlet fission time constants, and the S_1 dynamics correspond to the coherent singlet fission.

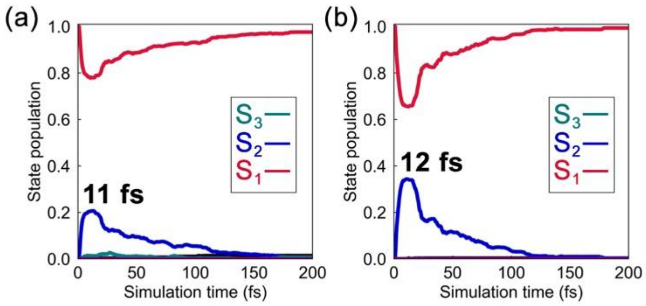

Supplementary Figure 10. State population dynamics from the transition-allowed S_1-FC points. (a) the herringbone and (b) parallel dimers in 200 fs ML-photodynamics simulations.

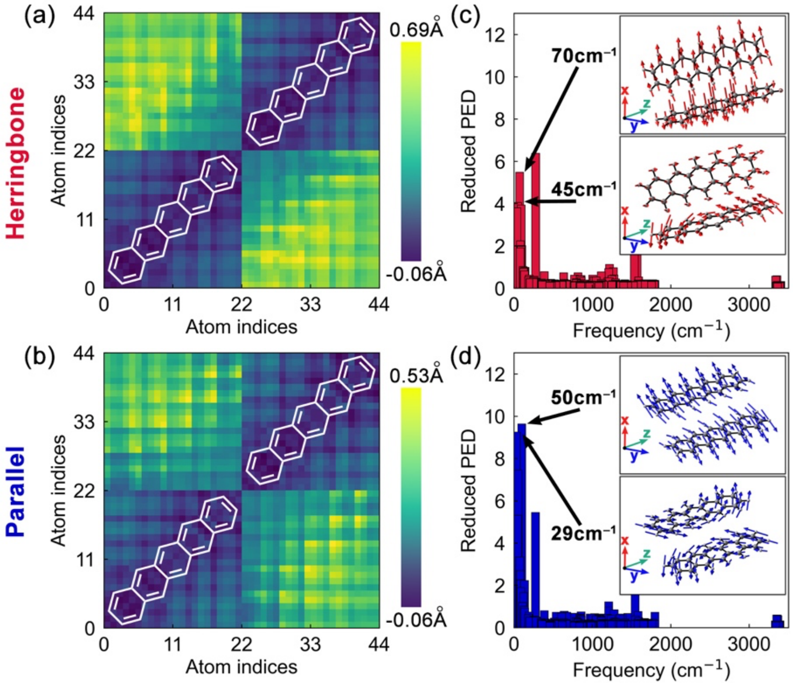

Supplementary Figure 11. Characterizations of pentacene dimer trajectories from the transition-allowed S_1-FC points. Differential Distance Matrices of (a) the herringbone and (b) parallel dimers based on the transition-allowed S_1-FC and final structures. Yellow corresponds to elongation; blue refers to shrink. Plots for the reduced potential energy distributions in the S_1 trajectories of (c) the herringbone and (d) parallel dimers with the two dominant vibrational modes. Detailed vibrational modes are available in Supplementary Figures 12 and 13.

Supplementary Note 6. Intermolecular vibrations

Supplementary Figures12 and S13 plot the complete reduced potential energy distributions with the dominant low-frequency vibrational modes for the S_2 trajectories of herringbone and parallel dimers, respectively.

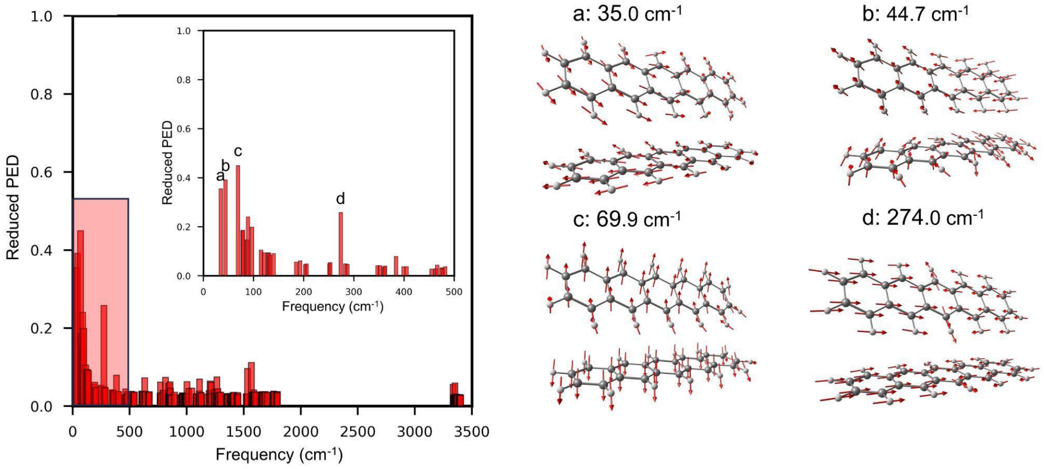

Supplementary Figure 12. Reduced potential energy distribution per normal mode in the S_2 trajectories for the herringbone dimer. The inset shows a zoomed view of the shaded region. The normal modes with the highest activity are also depicted. The vibrational modes are computed based on the S_1 minimum structure.

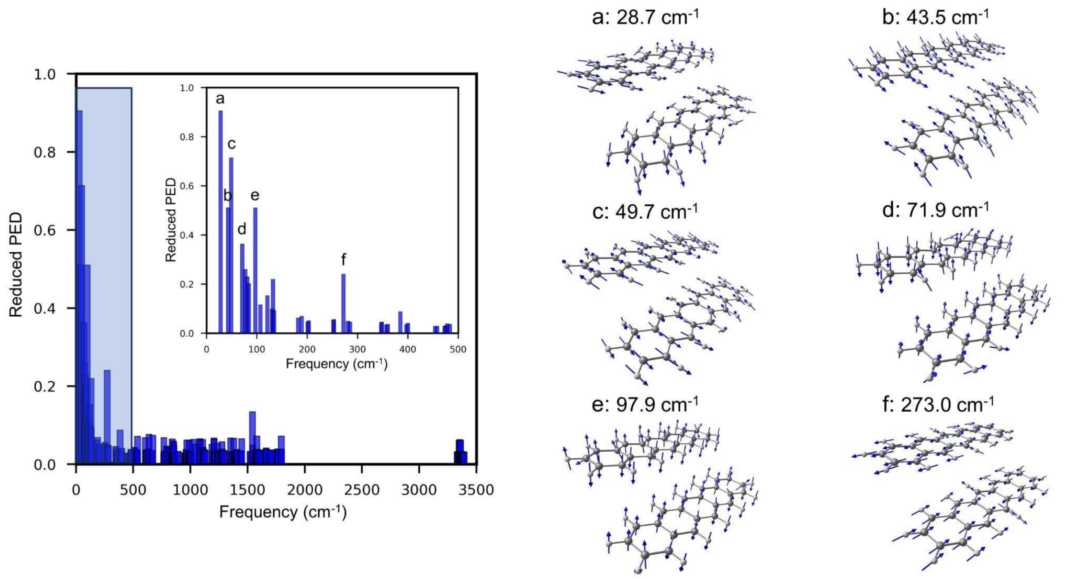

Supplementary Figure 13. Reduced potential energy distribution per normal mode in the S_2 trajectories for the parallel dimer. The inset shows a zoomed view of the shaded region. The normal modes with the highest activity are also depicted. The vibrational modes are computed based on the S_1 minimum structure.

Supplementary Note 7. Minimum energy conical intersections

We used the penalty function method[22] to optimize the S_2/S_1 MECIs as implemented in *fromage*. The UMAP of the S_2/S_1 surface hopping distributions in Figure 4 shows a broad distribution. We evenly sampled eight points as the guess structures for the S_2/S_1 MECI calculations, along with the edge of the clustering in the +x, +x+y, +y, y-x, -x, -x-y, -y, and x-y directions. The MECI optimizations converge to seven and eight geometries for the herringbone and parallel dimer respectively, shown in Supplementary Figure 14.

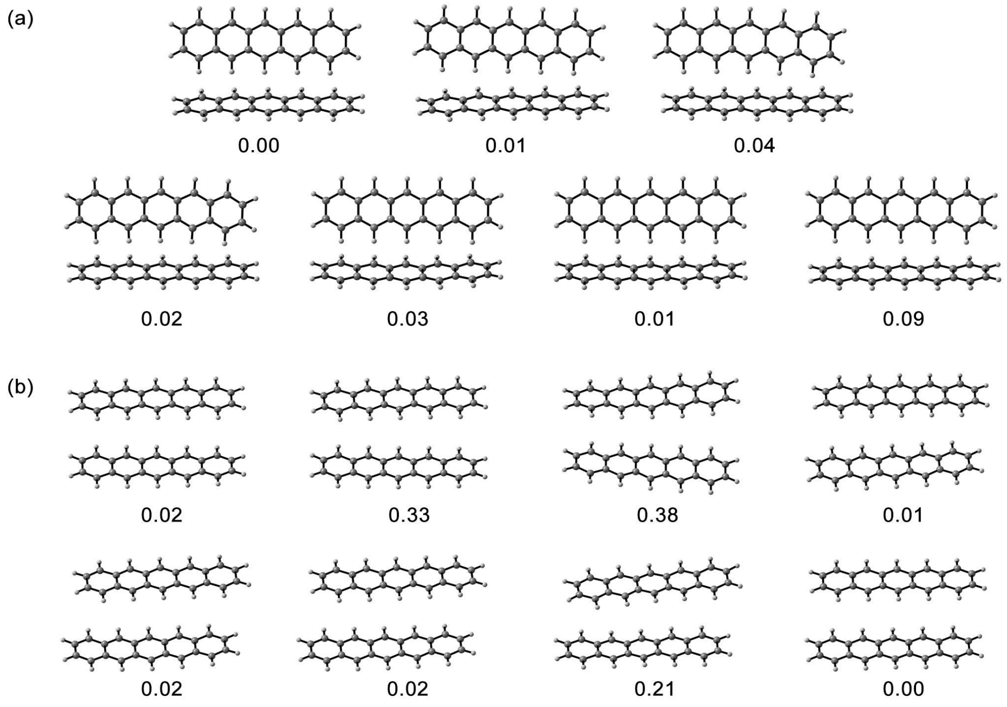

Supplementary Figure 14. Optimized S_2/S_1 minimum energy conical intersections structures with the SA6-CASSCF(4,4)/ANO-S-VDZP method. (a) the herringbone and (b)

the parallel dimer. Their relative electronic energies to the global minimum are shown under the structures in eV.

Supplementary Note 8. Rigid potential energy scans

In Figures 4c and 4d, the dominant vibrational modes in trajectories show the elongation of the intermolecular distances. However, the vibrational modes are also mixed with other motions, which makes it difficult to estimate the contributions of the intermolecular translations. To quantify the contributions from different intermolecular motions to the excited-state vibrations, we define four chemically intuitive motions in Supplementary Figure 15 corresponding to three orthogonal translations (M_x, M_y, M_z) and one rotation (M_r) of the monomers, where M_x follows the directions of the R1/R2 vectors.

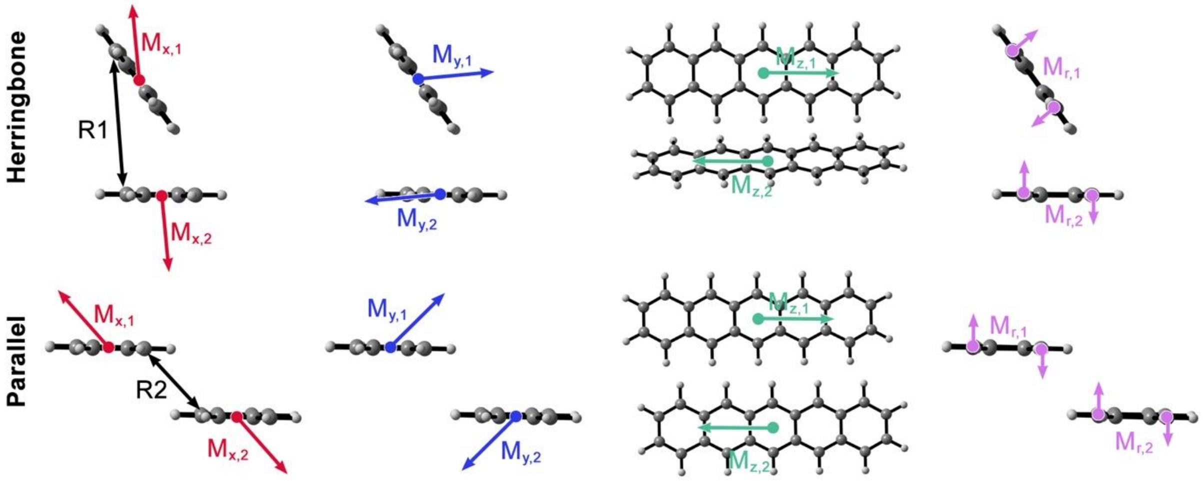

Supplementary Figure 15. Four chemically intuitive motions in the herringbone and parallel dimers.

We first project the vibrational modes that dominate the trajectories (Figures 4c and 4d) to the defined motions (Supplementary Figure 15). The projection norm represents the contributions of each motion.

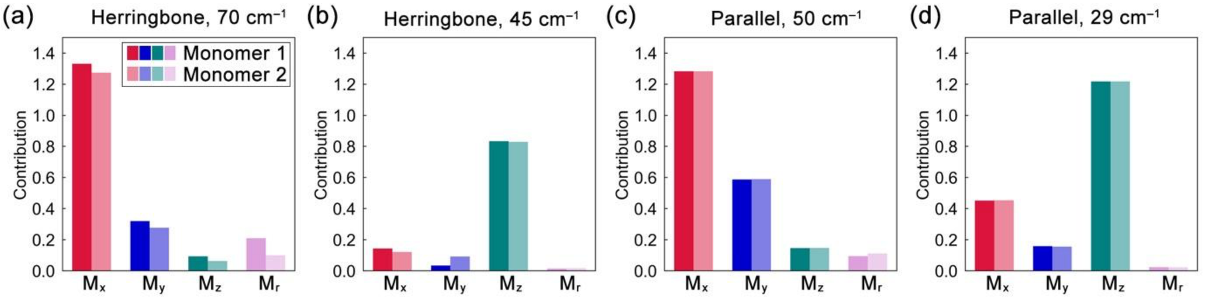

Supplementary Figure 16. Plots of the contributions from the defined motions to the dominant vibrational modes in the trajectories.

Supplementary Figures16a and S16b show substantial contributions from M_x and M_z to the 70 cm^{-1} and 45 cm^{-1} modes of the herringbone dimers, respectively. Supplementary Figures16c and S16d show M_x and M_z also dominate the 50 cm^{-1} and 29 cm^{-1} modes of the parallel dimers. These results suggest the elongation of intermolecular distance (M_x) and the lateral motions (M_z) are essential to understanding the $S_2 \rightarrow S_1$ decay mechanism.

Figures 4c and 4d in the main text indicate the participation of the other vibrational modes in the excited-state dynamics. To determine the total contributions of the defined motions in the dynamics, we computed the structural changes of the dimers at the end of the simulations from the initial conditions. We plotted their projection norm in Supplementary Figure 17. Our results show the trajectories of both herringbone and parallel dimers are governed by the elongation of the intermolecular distances (M_x). The contributions of M_y and M_z are comparable, while the rotations are minimal.

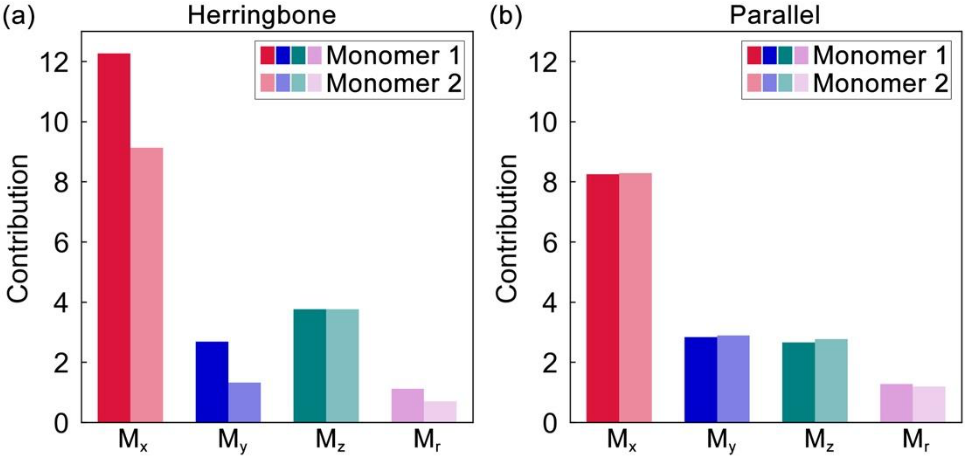

Supplementary Figure 17. Plots of the contributions from the defined motions to the structural changes in the trajectories.

Figure 5 revealed that the S_1 configurations change from CT to TT state with increasing distance along with M_x (i.e., R1/R2). Here, we performed rigid potential energy scan to investigate the relationships between the S_1 characters and the rest of the motions M_y, M_z, and M_r.

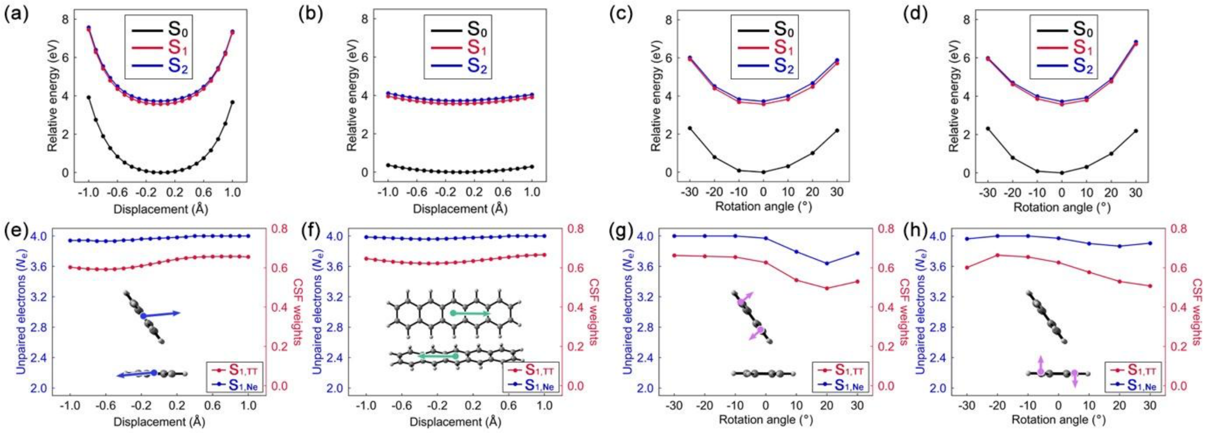

Supplementary Figure 18. Potential energy scans and electronic structure variations of the herringbone dimer. Plots for the potential energy curves of herringbone dimers following (a) M_y, (b) M_z, (c) M_r of monomer 1, and (d) M_r of monomer 2, computed with the ee-ONIOM(SA6-CASCI(4,4)/cc-pVDZ:GFN2-xTB). Plots for the number of unpaired electrons (N_e) and the weights of the TT state in the S_1 state of the herringbone dimers as functions of (e) M_y, (f) M_z, (g) M_r of monomer 1, and (h) M_r of monomer 2, where the corresponding motions are illustrated inside the figures.

Supplementary Figures18a-S18d illustrate the potential energy curves of the herringbone dimers displaced by M_y, M_z, and M_r of individual monomers. The S_0 minima structures are at the middle point of the scans. The S_2 and S_1 states are close-lying and show increasing energies following positive and negative displacements. These results suggest these motions are not responsible for the energy relaxations in S_2 and S_1 states. The potential energy curves following M_z are relatively flat (Supplementary Figure 18b), which explains the lateral motions observed in the dynamics. Supplementary Figures18e-S18h show the number of unpaired electrons (N_e) and the weights of the TT character in the S_1 state following the scan. M_y and M_z do not notably affect the unpaired electrons or the TT characters. The rotation of monomer 1 slightly reduced N_e and the TT character in the S_1 state when it is perpendicular to the other in Supplementary Figure 18g (M_r>0); the rotation of monomer 2 also decreased the TT characters in S_1 when the C-H bonds are rotating toward the benzene rings in Supplementary Figure 18h (M_r > 0). Nevertheless, the TT characters are still more than 50%, dominating the nature of the S_1 state.

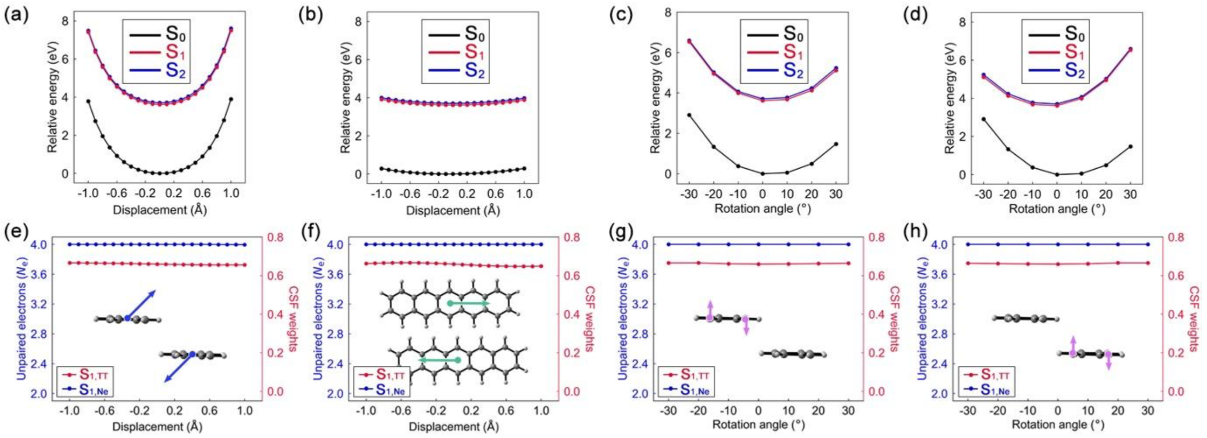

Supplementary Figure 19. Potential energy scans and electronic structure variations of the parallel dimer. Plots for the potential energy curves of parallel dimers following (a) M_y, (b) M_z, (c) M_r of monomer 1, and (d) M_r of monomer 2, computed with the ee-ONIOM(SA6-CASCI(4,4)/cc-pVDZ:GFN2-xTB). Plots for the number of unpaired electrons (N_e) and the weights of the TT character in the S_1 state of the parallel dimers as functions of (e) M_y, (f) M_z, (g) M_r of monomer 1, and (h) M_r of monomer 2, where the corresponding motions are illustrated inside the figures.

Supplementary Figure 19 plots the potential energy curves, N_e, and TT characters of the parallel dimers. The S_2 and S_1 energies show similar features as the herringbone dimers. The almost flat potential energy curves in M_z directions (Supplementary Figure 19b) explain the lateral motions observed in the dynamics. The N_e and TT characters remained nearly unchanged in all scans. Together with the results of the herringbone dimers (Supplementary Figure 18), our calculations suggest M_y, M_z, and M_r could not significantly affect the TT characters of the S_1 state. Therefore, the elongations of the intermolecular distances (M_x) are the driving force that generates the TT state in the pentacene dimers.

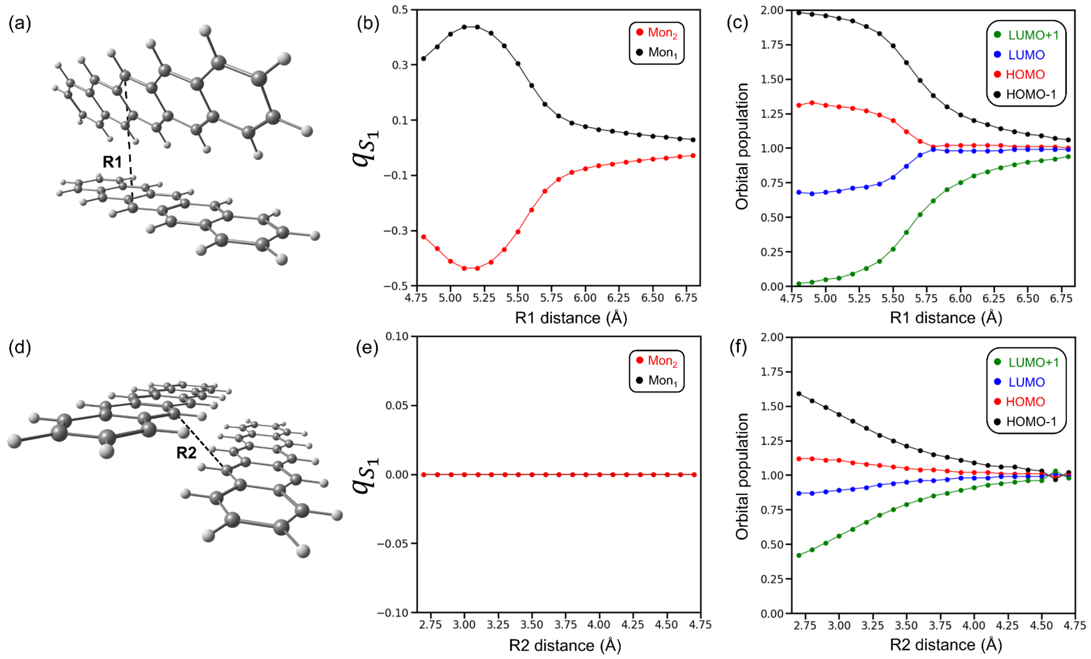

Supplementary Figure 20. Charge and orbital population scan of pentacene dimers. The excited-state scan of the pentacene dimer along with (a) the intermolecular distance R1 in the herringbone dimer and (b) R2 in the parallel dimer. The projected S_1 electronic charges on each monomer in (b) the herringbone and (e) parallel dimer. The active orbital populations in the S_1 state for (c) the herringbone and (f) parallel dimer, computed at the SA6-CASSCF(4,4)/ANO-S-VDZP level of theory. The active orbitals are labeled according to their order in the DFT calculations.

Supplementary Note 9. Notes on the crystal environment

We computed the Voronoi volume and the van der Waals (vdW) volume for each pentacene monomer to estimate the maximum available space inside the crystal and the occupied space, respectively. The ratio between Voronoi and van der Waals volume, called the volume index (Vi), shows the extent of the molecular flexibility. The Vi of the optimized structure in the herringbone and parallel dimer is 1.43 and 1.42, respectively. These values suggest large volumes available for the excited-state vibrations. Moreover, we performed the ML photodynamics simulations with a flexible crystal environment to assess the influence of the excited-state vibrations and dynamics. We chose the GFN-FF for computing the crystal environment because of its similar performance to GFN2-xTB and high efficiency. Supplementary Table 5 collects the initial and final average Voronoi, vdW volume, and Vi of both dimers in the ML photodynamics simulations with rigid (NN/GFN2-xTB, NN/GFN-FF, and NN/GFN-FF) and flexible crystal environment (NN/GFN-FF).

Supplementary Table 5. Calculated Voronoi and van der Waals volume with volume index for various models. The values in the parenthesis indicate the standard deviations.

Dimers	Model	Voronoi (Å^3)	vdW (Å^3)	Vi
Herringbone	Initial	350.50(2.76)	249.76(1.60)	1.40
	NN/GFN2-xTB	360.33(2.75)	251.17(1.67)	1.43
	NN/GFN-FF(rigid)	358.24(2.81)	250.91(1.71)	1.43
	NN/GFN-FF(flexible)	362.60(3.20)	250.80(1.71)	1.45
Parallel	Initial	350.85(3.66)	250.40(1.70)	1.40
	NN/GFN2-xTB	355.01(3.21)	251.12(1.57)	1.41
	NN/GFN-FF(rigid)	352.25(3.29)	250.96(1.65)	1.40
	NN/GFN-FF(flexible)	356.17(4.11)	251.12(1.69)	1.42

At the end of simulations, the pentacene monomers in the herringbone dimers show slightly larger increments of the Voronoi volume in the flexible crystal environments (dV≈12Å^3) than in the rigid crystal environments (dV≈10Å^3). The monomers in the parallel dimers show comparable changes in the Voronoi volumes in the flexible (dV≈ 5Å^3) and rigid (dV≈4Å^3)crystal environments. The vdW volumes in both dimers show small changes of less than 2Å^3. As a result, the Vi in both dimers is almost unchanged regardless of whether the crystal environment is fixed, suggesting that the pentacene crystal provides sufficient volume for the excited-state vibrations in the subpicosecond timescale.

We further compared the trajectories from the S_2-FC regions with rigid and flexible crystal environments. The $S_2 \rightarrow S_1$ decay time constants for the herringbone dimers are 61, 69, and 69 fs, obtained by the NN/GFN2-xTB, NN/GFN-FF, and NN/GFN-FF(flexible) models, respectively. For the parallel dimers, the $S_2 \rightarrow S_1$ decay time constants are 33, 40, and 43 fs, respectively, obtained by the NN/GFN2-xTB, NN/GFN-FF, and NN/GFN-FF(flexible) models, respectively. We plot the trajectories in Supplementary Figure 21, where the black curves represent the averaged trajectories. The results of NN/GFN-F in Supplementary Figure 21e-

S21h show the same structural changes as the NN/GFN-FF(flexible) in Supplementary Figures21i-S21l.

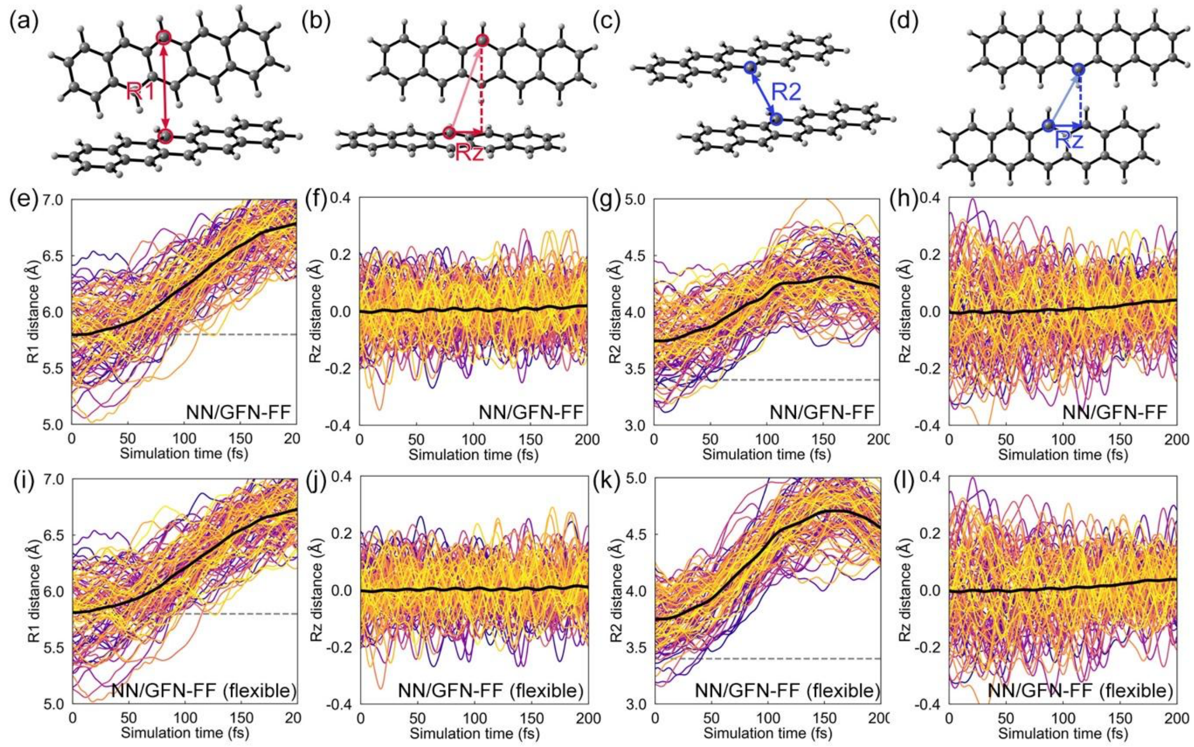

Supplementary Figure 21. Benchmarks of the trajectories. (a)-(d) Illustrations of the intermolecular distances and lateral motions used to track the trajectories of the herringbone and parallel dimers. Plots for 100 randomly selected S_2 trajectories of the herringbone and parallel dimers obtained with the NN/GFN-FF method in (e)-(h) a rigid crystal environment and (i)-(l) a flexible crystal environment.

Supplementary Note 10. Data and code availability

The fromage code is open-source and released at:
https://github.com/Crespo-Otero-group/fromage

The PyRAI^{2}MD code is open-sourced and released at:
https://github.com/mlcclab/PyRAI2MD-hiam. DOI: 10.5281/zenodo.14546617.

The NN models, a small set of initial conditions and input files are available at:
https://github.com/mlcclab/PyRAI2MD_publications/tree/main/Pentacene_dimers

Full training data, initial conditions and all trajectory data are available in Figshare, DOI: 10.6084/m9.figshare.28082003.
Source data are provided with this paper.

Supplementary reference

1. Sahar Sharifzadeh, S.; Pierre Darancet, P.;Leeor Kronik, L.; Neaton, J. B. Low-Energy Charge-Transfer Excitons in Organic Solids from First-Principles: The Case of Pentacene. *J. Phys. Chem. Lett.* **2013**, *4*, 13, 2197–2201. https://doi.org/10.1021/jz401069f.

2. Duan. H,; JhA, A.; Li, X.; Tiwari, V.; Ye, H.; Nayak, P.; Zhu, X.; Li, Z.; Martinez, T. J.; Thorward,; M.; Miller, R.J. D. Intermolecular vibrations mediate ultrafast singlet fission. *Sci. Adv.* **2020**. *6*, eabb0052. https://doi.org/10.1126/sciadv.abb0052
3. Refaely-Abramson, S.; da Jornada, F. H.; Louie, S. G.; Neaton, J. B. Origins of Singlet Fission in Solid Pentacene from an ab initio Green's Function Approach. *Phys. Rev. Lett.* **2017,** *119*, 26740. https://doi.org/10.1103/PhysRevLett.119.267401.
4. Rivera, M.; Dommett, M.; Sidat, A.; Rahim, W.; Crespo-Otero, R. Fromage: A Library for the Study of Molecular Crystal Excited States at the Aggregate Scale. *Journal of Computational Chemistry* **2020**, *41* (10), 1045–1058. https://doi.org/10.1002/jcc.261445.
5. Frisch, M. J.; Trucks, G. W.; Schlegel, H. B.; Scuseria, G. E.; Robb, M. A.; Cheeseman, J. R.; Scalmani, G.; Barone, V.; Petersson, G. A.; Nakatsuji, H.; Li, X.; Caricato, M.; Marenich, A. V.; Bloino, J.; Janesko, B. G.; Gomperts, R.; Mennucci, B.; Hratchian, H. P.; Ortiz, J. V.; Izmaylov, A. F.; Sonnenberg, J. L.; Williams-Young, D.; Ding, F.; Lipparini, F.; Egidi, F.; Goings, J.; Peng, B.; Petrone, A.; Henderson, T.; Ranasinghe, D.; Zakrzewski, V. G.; Gao, J.; Rega, N.; Zheng, G.; Liang, W.; Hada, M.; Ehara, M.; Toyota, K.; Fukuda, R.; Hasegawa, J.; Ishida, M.; Nakajima, T.; Honda, Y.; Kitao, O.; Nakai, H.; Vreven, T.; Throssell, K.; Montgomery, J. A., Jr.; Peralta, J. E.; Ogliaro, F.; Bearpark, M. J.; Heyd, J. J.; Brothers, E. N.; Kudin, K. N.; Staroverov, V. N.; Keith, T. A.; Kobayashi, R.; Normand, J.; Raghavachari, K.; Rendell, A. P.; Burant, J. C.; Iyengar, S. S.; Tomasi, J.; Cossi, M.; Millam, J. M.; Klene, M.; Adamo, C.; Cammi, R.; Ochterski, J. W.; Martin, R. L.; Morokuma, K.; Farkas, O.; Foresman, J. B.; Fox, D. J. 16 Revision C.01, 2016.
6. Bannwarth, C.; Ehlert, S.; Grimme, S. GFN2-xTB—An Accurate and Broadly Parametrized Self-Consistent Tight-Binding Quantum Chemical Method with Multipole Electrostatics and Density-Dependent Dispersion Contributions. *J. Chem. Theory Comput.* **2019**, *15* (3), 1652–1671. https://doi.org/10.1021/acs.jctc.8b01176.
7. Fdez Galván, I.; Vacher, M.; Alavi, A.; Angeli, C.; Aquilante, F.; Autschbach, J.; Bao, J. J.; Bokarev, S. I.; Bogdanov, N. A.; Carlson, R. K.; Chibotaru, L. F.; Creutzberg, J.; Dattani, N.; Delcey, M. G.; Dong, S. S.; Dreuw, A.; Freitag, L.; Frutos, L. M.; Gagliardi, L.; Gendron, F.; Giussani, A.; González, L.; Grell, G.; Guo, M.; Hoyer, C. E.; Johansson, M.; Keller, S.; Knecht, S.; Kovačević, G.; Källman, E.; Li Manni, G.; Lundberg, M.; Ma, Y.; Mai, S.; Malhado, J. P.; Malmqvist, P. Å.; Marquetand, P.; Mewes, S. A.; Norell, J.; Olivucci, M.; Oppel, M.; Phung, Q. M.; Pierloot, K.; Plasser, F.; Reiher, M.; Sand, A. M.; Schapiro, I.; Sharma, P.; Stein, C. J.; Sørensen, L. K.; Truhlar, D. G.; Ugandi, M.; Ungur, L.; Valentini, A.; Vancoillie, S.; Veryazov, V.; Weser, O.; Wesołowski, T. A.; Widmark, P.-O.; Wouters, S.; Zech, A.; Zobel, J. P.; Lindh, R. OpenMolcas: From Source Code to Insight. *J Chem Theory Comput* **2019**, *15* (11), 5925–5964. https://doi.org/10.1021/acs.jctc.9b00532.
8. Shiozaki, T. BAGEL: Brilliantly Advanced General Electronic-Structure Library. *WIREs Computational Molecular Science* **2018**, *8* (1), e1331. https://doi.org/10.1002/wcms.1331.
9. Li, J.; Reiser, P.; Boswell, B. R.; Eberhard, A.; Burns, N. Z.; Friederich, P.; Lopez, S. A. Automatic Discovery of Photoisomerization Mechanisms with Nanosecond Machine Learning Photodynamics Simulations. *Chem. Sci.* **2021**, *12* (14), 5302–5314. https://doi.org/10.1039/D0SC05610C.
10. Zimmerman, P. M.; Bell, F.; Casanova, D.; Head-Gordon, M. Mechanism for Singlet Fission in Pentacene and Tetracene: From Single Exciton to Two Triplets. *J. Am. Chem. Soc.* **2011**, *133* (49), 19944–19952. https://doi.org/10.1021/ja208431r.
11. Horbatenko, Y.; Sadiq, S.; Lee, S.; Filatov, M.; Choi, C. H. Mixed-Reference Spin-Flip Time-Dependent Density Functional Theory (MRSF-TDDFT) as a Simple yet Accurate Method for Diradicals and Diradicaloids. *J. Chem. Theory Comput.* **2021**, *17*, 848–859. https://doi.org/10.1021/acs.jctc.0c01074.
12. Mironov, V.; Komarov, K.; Li, J.; Gerasimov, I.; Nakata, H.; Mazaheri, M. Ishimura, K.; Park, W.; Lashkaripour, A.; Oh, M.; Huix-Rotllant, M.; Lee, S.; Choi, C.H. MRSF-TDDFT with an Emphasis on Open-source Ecosystem. J. Chem. Theory Comput., **2024**, 2024, 20, 9464–9477. https://doi.org/ 10.1021/acs.jctc.4c01117.

13. Zimmerman, P.; Zhang, Z.; Musgrave, C. Singlet fission in pentacene through multi-exciton quantum states. *Nat. Chem.* **2010**, *2*, 648–652. https://doi.org/10.1038/nchem.694.
14. Lu, T. A comprehensive electron wavefunction analysis toolbox for chemists, Multiwfn. *J. Chem. Phys.* **2024**, 161, 082503. https://doi.org/10.1063/5.0216272.
15. Abadi, M.; Agarwal, A.; Barham, P.; Brevdo, E.; Chen, Z.; Citro, C.; Corrado, G.; Davis, A.; Dean, J.; Devin, M.; Ghemawat, S.; Goodfellow, I.; Harp, A.; Irving, G.; Isard, M.; Jia, Y.; Józefowicz, R.; Kaiser, L.; Kudlur, M.; Levenberg, J.; Mané, D.; Monga, R.; Moore, S.; Murray, D.; Olah, C.; Schuster, M.; Shlens, J.; Steiner, B.; Sutskever, I.; Talwar, K.; Tucker, P.; Vanhoucke, V.; Vasudevan, V.; Viégas, F.; Vinyals, O.; Warden, P.; Wattenberg, M.; Wicke, M.; Yu, Y.; Zheng, X. TensorFlow: Large-Scale Machine Learning on Heterogeneous Distributed Systems. *ArXiv* **2016**.
16. Shu, Y.; Zhang, L.; Chen, X.; Sun, S.; Huang, Y.; Truhlar, D. G. Nonadiabatic Dynamics Algorithms with Only Potential Energies and Gradients: Curvature-Driven Coherent Switching with Decay of Mixing and Curvature-Driven Trajectory Surface Hopping. *J. Chem. Theory Comput.* **2022**, *18* (3), 1320–1328. https://doi.org/10.1021/acs.jctc.1c01080.
17. Zhao, X.; Merritt, I. C. D.; Lei, R.; Shu, Y.; Jacquemin, D.; Zhang, L.; Xu, X.; Vacher, M.; Truhlar, D. G. Nonadiabatic Coupling in Trajectory Surface Hopping: Accurate Time Derivative Couplings by the Curvature-Driven Approximation. *J. Chem. Theory Comput.* **2023**, *19* (19), 6577–6588. https://doi.org/10.1021/acs.jctc.3c00813.
18. Baeck, K. K.; An, H. Practical Approximation of the Non-Adiabatic Coupling Terms for Same-Symmetry Interstate Crossings by Using Adiabatic Potential Energies Only. *The Journal of Chemical Physics* **2017**, *146* (6), 064107. https://doi.org/10.1063/1.4975323.
19. Kim, J.; Bain, D.C.; Ding, V.; Majumder. K.; Windemuller, D.; Feng, J.; Wu, J.; Patil, S.; Anthony, J.; Kim, W.; Musser, A. J. Coherent photoexcitation of entangled triplet pair states. Nat. Chem. **2024**, *in press.* https://doi.org/10.1038/s41557-024-01556-3
20. Tully, J. C. Molecular Dynamics with Electronic Transitions. *The Journal of Chemical Physics* **1990**, *93* (2), 1061–1071. https://doi.org/10.1063/1.459170.
21. Hammes-Schiffer, S.; Tully, J. C. Proton Transfer in Solution: Molecular Dynamics with Quantum Transitions. *The Journal of Chemical Physics* **1994**, *101* (6), 4657–4667. https://doi.org/10.1063/1.467455.
22. Levine, B. G.; Coe, J. D.; Martínez, T. J. Optimizing Conical Intersections without Derivative Coupling Vectors: Application to Multistate Multireference Second-Order Perturbation Theory (MS-CASPT2). *J. Phys. Chem. B* **2008**, *112* (2), 405–413. https://doi.org/10.1021/jp0761618.
